# Optical Methods for Non-Invasive Determination of Skin Penetration: Current Trends, Advances, Possibilities, Prospects, and Translation into In Vivo Human Studies

**DOI:** 10.3390/pharmaceutics15092272

**Published:** 2023-09-03

**Authors:** Maxim E. Darvin

**Affiliations:** Independent Researcher, 10178 Berlin, Germany; maxim.darvin@protonmail.com

**Keywords:** Raman micro-spectroscopy, coherent Raman, laser scanning microscopy, two-photon tomography, three-photon tomography, fluorescence lifetime imaging, drug delivery, optical coherence tomography, skin barrier, tape stripping

## Abstract

Information on the penetration depth, pathways, metabolization, storage of vehicles, active pharmaceutical ingredients (APIs), and functional cosmetic ingredients (FCIs) of topically applied formulations or contaminants (substances) in skin is of great importance for understanding their interaction with skin targets, treatment efficacy, and risk assessment—a challenging task in dermatology, cosmetology, and pharmacy. Non-invasive methods for the qualitative and quantitative visualization of substances in skin in vivo are favored and limited to optical imaging and spectroscopic methods such as fluorescence/reflectance confocal laser scanning microscopy (CLSM); two-photon tomography (2PT) combined with autofluorescence (2PT-AF), fluorescence lifetime imaging (2PT-FLIM), second-harmonic generation (SHG), coherent anti-Stokes Raman scattering (CARS), and reflectance confocal microscopy (2PT-RCM); three-photon tomography (3PT); confocal Raman micro-spectroscopy (CRM); surface-enhanced Raman scattering (SERS) micro-spectroscopy; stimulated Raman scattering (SRS) microscopy; and optical coherence tomography (OCT). This review summarizes the state of the art in the use of the CLSM, 2PT, 3PT, CRM, SERS, SRS, and OCT optical methods to study skin penetration in vivo non-invasively (302 references). The advantages, limitations, possibilities, and prospects of the reviewed optical methods are comprehensively discussed. The ex vivo studies discussed are potentially translatable into in vivo measurements. The requirements for the optical properties of substances to determine their penetration into skin by certain methods are highlighted.

## 1. Introduction

The precise delivery of particulate and non-particulate active pharmaceutical ingredients (APIs) and functional cosmetic ingredients (FCIs) in skin is a challenging task in dermatology, cosmetology, and pharmacy that requires an advanced understanding of the skin barrier structure. Some formulations are designed to permeate the *stratum corneum* (SC) or even the entire epidermis to reach their targets in the viable epidermis or dermis (e.g., drugs), while other formulations are intended to stay exclusively on the skin surface (e.g., sunscreens) or to remain throughout the SC (e.g., some drugs or cosmetics) [1,2]. To ensure deep penetration of the APIs into the skin, the SC integrity should be locally disrupted, which is usually achieved by adding penetration enhancers—active chemical vehicles—to the drug formulations [3,4]. For instance, the functional nano-systems containing penetration enhancers are promising for targeted transdermal drug delivery [5]. Air pollutants may penetrate and accumulate in the skin (mainly present in particulate matter of nm–µm-range [6]) and have a negative influence on the skin, and are, therefore, not desirable [7,8,9,10]. The penetration depth is increased in barrier-damaged skin [11]. 

**Figure 1:**
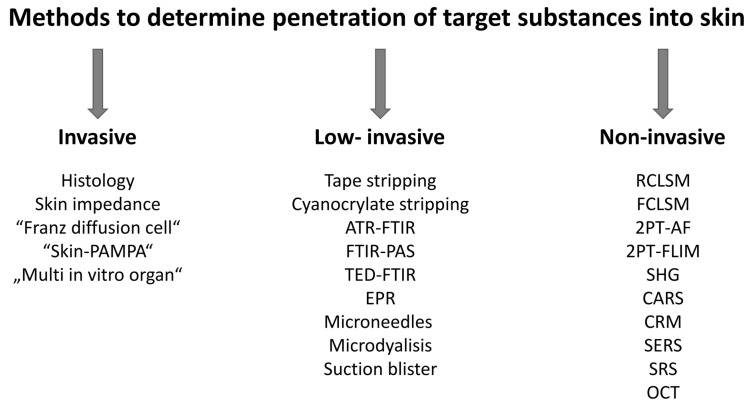
Schematic illustration of methods, subdivided into invasive, low-invasive and non-invasive, used to determine penetration depth of target substances into skin.

The determination of the penetration depth of vehicles, APIs, and FCIs of topically applied formulations or contaminants (further called “target substances” or “substances”) into the skin is of great importance for understanding their interaction with targets, treatment efficacy, and risk assessment. The modelling [12] and prediction [13] of skin penetration are complicated, and experimental methods are required. Although the most relevant way to determine skin penetration is in vivo human studies [14], existing regulations and guidelines for human research force us to perform investigations on animal skin and artificial skin models [14,15]. The existing methods for determining skin penetration can generally be divided into low-invasive, invasive, and non-invasive (Figure 1). 

Low-invasive methods can be applied both in vivo and ex vivo and include tape stripping, primarily applied at the SC [16,17,18]; cyanoacrylate stripping applicable to the SC and viable epidermis [18,19]; suction blister after the application of partial negative pressure on the skin with further analysis of the accumulated interstitial and serum fluids [20]; sampling of the interstitial fluid from the viable epidermis and/or dermis with microneedles [21,22,23] or by microdialysis [24,25], followed by an analysis with validated methods (or their combinations) such as autoradiography for the detection of the radioisotope-labelled penetrants [26], high-performance liquid chromatography, mass spectrometry of various modifications, or UV/VIS spectroscopy (absorption or (epi)fluorescence in a specific spectral range) [26,27,28,29,30]. The skin penetration profile can be determined using low-invasive attenuated total reflectance Fourier-transform infrared (ATR-FTIR) [31,32,33], Fourier-transform infrared photoacoustic spectroscopy (FTIR-PAS) [31], thermal emission decay–Fourier-transform infrared (TED-FTIR) spectroscopy [34], and electron paramagnetic resonance (EPR) spectroscopy [35] for the analysis of the SC removed from skin with tape or cyanoacrylate stripping. The degree of invasiveness varies among the low-invasive methods: for example, tape stripping is the least invasive procedure, while suction blister and microdialysis are the most invasive [20]. Tape-stripping-based low-invasive ATR-FTIR and EPR spectroscopy methods are commonly used in in vivo skin penetration studies, but require special permission and are severely limited in patients. 

Invasive methods always involve taking a skin biopsy. The “Franz diffusion cell” [36,37,38], “skin-PAMPA” (skin parallel artificial membrane permeability assay) [39,40,41], and “multi in vitro organ” [42] methods can be used, in addition to conventional histology, for in vitro and ex vivo studies of penetration using artificial membranes, skin models, or skin biopsies [43]. Target substances can be determined using the methods described above for the low-invasive analysis. To image the particulate substances in the skin biopsy, transmission electron microscopy, scanning electron microscopy, atomic force microscopy, cryo-electron microscopy, and soft X-ray spectro-microscopy can be used in vitro and ex vivo [44,45]. Fluorescence microscopy enables selective staining and requires thin skin specimens (5–10 µm), as scattered light from thicker samples interferes with the detection of the primary signal, resulting in blurry images [46]. The use of invasive methods is strictly regulated due to ethical constraints and local laboratory regulations. Invasive methods are used for skin penetration analysis in vitro and ex vivo and are described in detail elsewhere [20,26,47,48,49].

In general, it is important to consider that the sampling of the SC and/or skin biopsy (which is then analyzed ex vivo for the presence of target substances using low-invasive or invasive methods) leads to an intensified interaction with air, potentially causing the oxidation of skin constituents and the target substances; the increased evaporation of water from the sample and further water redistribution, resulting in the deformation of skin cells and loss of overall skin volume, firmness, and elasticity, as well as skin disinfection prior to biopsy collection; a skin biopsy sample temperature of 19–22 °C, which is lower than the skin temperature in vivo (30–32 °C); the preparation of the skin sample using chemicals; and storage in the freezer—all these factors may impair the skin barrier function and affect the results of the penetration analysis, which may differ from those measured under native in vivo conditions. In addition, taking a skin biopsy is a highly invasive minor surgical procedure that requires medical personnel and ethical approval and may affect the cosmetic appearance of the skin after recovery, i.e., not desired by the volunteers. Therefore, non-invasive methods are advantageous for in vivo application and can be used for the quantitative and qualitative determination of the penetration of target substances into the skin under native conditions—an “optical biopsy” can be performed non-invasively. Non-invasive methods are limited to optical methods, such as reflectance/fluorescence confocal laser scanning microscopy (RCLSM/FCLSM); two-photon tomography (2PT) combined with autofluorescence (2PT-AF), fluorescence lifetime imaging (2PT-FLIM), second-harmonic generation (SHG), coherent anti-Stokes Raman scattering (CARS), and reflectance confocal microscopy (RCM); confocal Raman micro-spectroscopy (CRM); surface-enhanced Raman scattering (SERS) micro-spectroscopy; stimulated Raman scattering (SRS) microscopy; and optical coherence tomography (OCT). 

This review summarizes the state of the art in the use of optical non-invasive methods in the study of skin penetration in vivo and discusses their advantages, limitations, and prospects. Many studies conducted ex vivo are also discussed as they are potentially translatable into in vivo measurements.

## 2. Skin Barrier Function and Penetration Pathways

**Figure 2:**
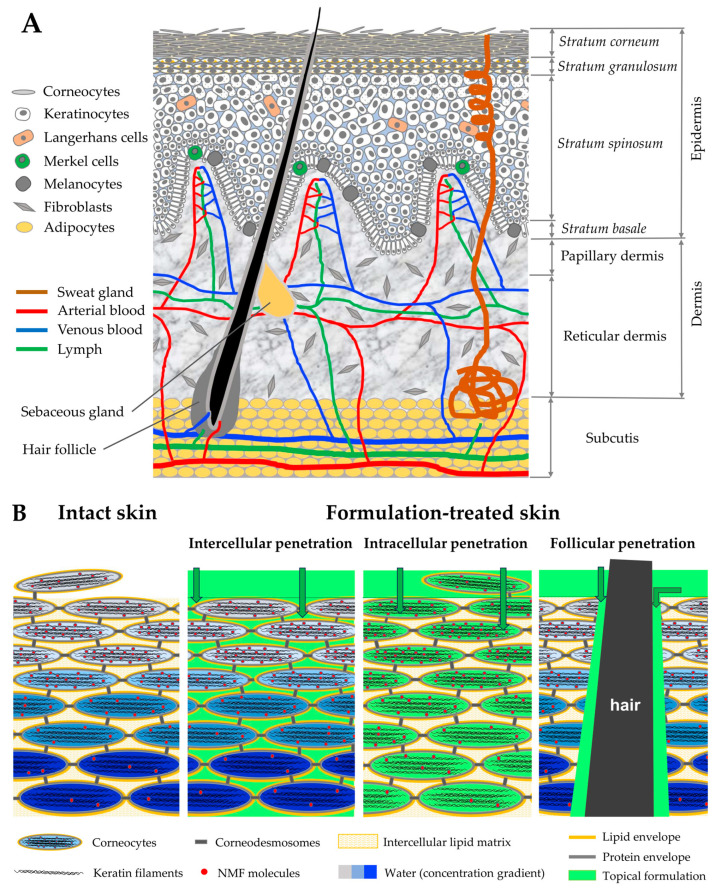
Schematic representation of hairy skin (**A**) and the SC structure of intact skin with three distinct penetration pathways (intercellular, intracellular, and follicular) of the topically applied formulation (green) (**B**). Arrows show the penetration directions.

The skin is a heterogeneous multifunctional organ with a layered structure divided into the epidermis, dermis, and subcutis. The epidermis is free of blood and lymph circulation, which is first found in the papillary dermis and deeper (Figure 2A). The upper epidermal layers—the SC and *stratum granulosum*—are mainly responsible for maintaining the skin barrier function [50]. In the SC, corneocytes (typically 15–20 layers), surrounded by the hard-permeable lipid and protein envelopes, are embedded in the low-permeable structural lipid bilayer matrix with a high content of orthorhombic lateral organization (ordered, very densely packed lipids), which are crucial for maintaining the primary physical barrier of the skin [50,51,52]. Below the SC, in the viable epidermis, tight junction proteins form a hard-permeable intercellular network that is most pronounced in the *stratum granulosum* and then decreases towards the *stratum basale*, forming the secondary physical barrier of the skin [53,54]. The basement membrane itself, localized at the basal side of the *stratum basale* and composed of a dense mesh of structural proteins and carbohydrates, represents the final barrier before the exogenous substance enters the blood and lymph microcirculation in the papillary dermis and the further systemic circulation [55]. This entire barrier acts efficiently against water and electrolyte losses from inside the body and restricts the penetration of contaminants (xenobiotics and pathogens) and topically applied formulations from outside the body. 

When applied topically, formulations can penetrate the skin by passive diffusion via three distinct and independent pathways: intercellularly (predominant), intracellularly (negligible), and through the skin appendages (limited role for hair follicles and sweat glands) [56,57,58,59,60], which are schematically shown in Figure 2B. Sweat glands mainly provide the inverse penetration pathway from the inside to the outside by releasing substances on the skin surface [57] (known as inside–outside penetration [6]). The molecular weight-based rule was proposed by Bos et al. [61] for the intercellular penetration of chemical compounds and drugs through the SC of normal skin, where only substances below 500 Daltons with moderate lipophilicity (log *P* 1–3) and aqueous solubility (>1 mg/mL) permeate the SC [62]. In skin diseases (e.g., atopic dermatitis) and barrier-disrupted skin, substances with a higher molecular weight can permeate the SC. Particles with a size of ≈650 nm penetrate deeper into the hair follicles after a skin massage than smaller or larger particles [63]. Thus, the skin barrier integrity [11]; the target substance/formulation-specific multiple characteristics, such as its composition, solubility, hydrogen-bonding groups, steric interaction of hydroxyl groups, viscosity, ionization, log *P*, and vehicle form (non-particulate/particulate, and the size of particles and aggregates) [20,64,65]; and penetration enhancement (chemical penetration enhancers [66], and physical procedures—thermal ablation, electroporation, iontophoresis, jet injection, ultrasound, and microneedles [67,68]) all affect the kinetics and depth of penetration into the skin.

**Figure 3:**
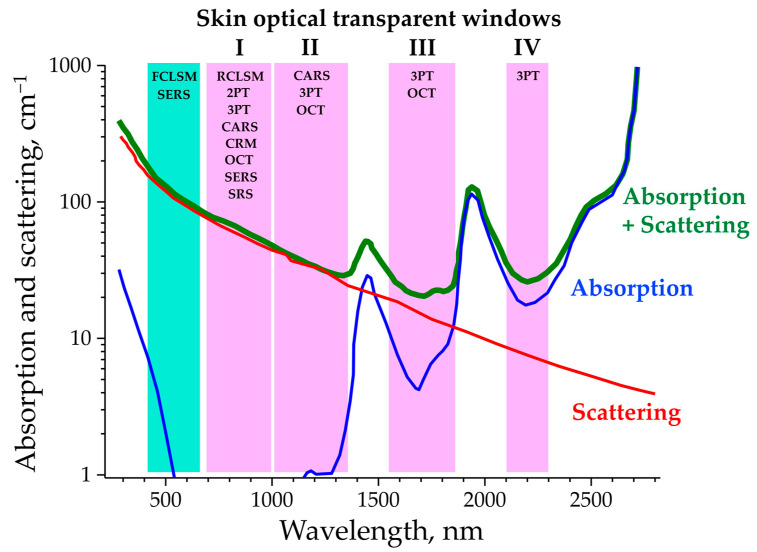
Representative absorption (blue line), scattering (red line), and combined absorption and scattering (green line) spectra of human Caucasian skin in the wavelength range of 280 to 2800 nm. The spectra in the optical ranges of 280–800 and 800–2800 nm are adopted under CC BY 4.0 license from Refs. [70,71], respectively. The optical transparent (therapeutic) windows of the skin (I–IV) are highlighted in pink; the non-transparent blue–red spectral range is highlighted in turquoise. The excitation wavelengths used in the optical methods (FCLSM, RCLSM, 2PT, 3PT, CARS, CRM, SERS, SRS, and OCT) are in the corresponding spectral range.

Figure 3 shows the characteristic absorption, scattering, and combined absorption and scattering spectra of human Caucasian skin in the broad spectral range of 280–2800 nm, which is crucial for choosing the appropriate optical method when imaging skin and studying skin penetration. The four skin optical transparent (therapeutic) windows are defined as I (≈700–1000 nm); II (≈1000–1350 nm); III (≈1550–1870 nm) and IV (≈2100–2300 nm), according to [69]. The methods described below can be used in vivo or ex vivo to non-invasively determine topically applied formulations (soluble or particulate), their vehicle, APIs, FCIs, or pollutants that have penetrated the skin intercellularly, intracellularly, and, with some limitations, follicularly, providing information about the penetration depth profile and pathway. The optical working range of the methods is illustrated in Figure 3.

## 3. Confocal Laser Scanning Microscopy (CLSM)

**Figure 4:**
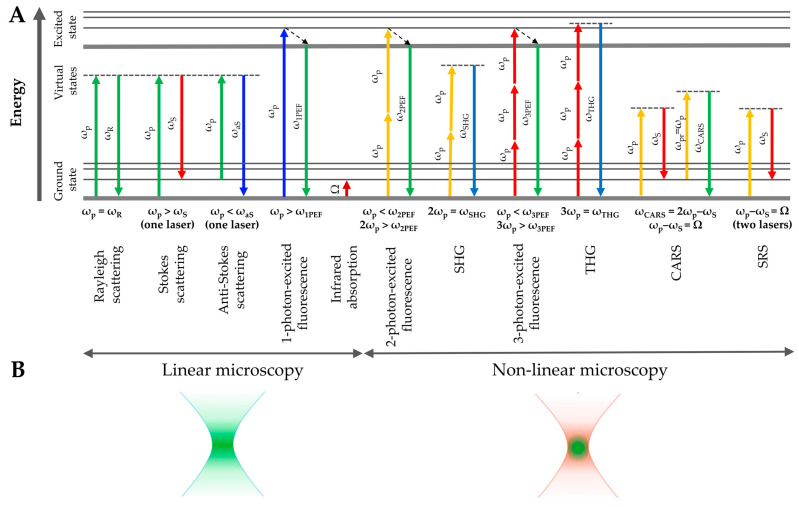
Jablonski diagram of linear and non-linear optical processes (**A**) and spatial localization of the signal with linear (the signal is generated in the entire volume) and non-linear (the signal is generated only in a small femtoliter volume) excitation. The distribution of light energy is shown in the green color gradient (**B**). The colors of the arrows in (**A**) indicate the shift (increase or decrease) of the emission wavelength in relation to the excitation wavelength (for a single process). The figures are adopted with permission from Ref. [76].

CLSM is a 3D optical imaging method in which fluorescence is excited by a collimated continuous-wave laser. The energy of the illumination photon (usually in the visible spectral range) is sufficient for bringing the fluorophore molecule into the excited state; then, the emitted one-photon-excited fluorescence is measured (Figure 4A—linear microscopy—shows the process schematically). Then, scanning mirrors or an objective scan of the laser beam across the sample was carried out to produce a horizontal image. Depth scanning is performed by moving the focus within the sample. Since the fluorophores are also excited outside the focal plane (Figure 4B), confocality is achieved by using a pinhole to block photons from outside the focal plane. Thus, only photons excited in the focal plane produce an intensity image. The reflection signal can also be collected. The working principle of the CLSM is described in detail elsewhere [72,73,74,75].

### 3.1. CLSM in Skin Morphology Imaging

CLSM is widely used in in vivo and ex vivo skin research [77,78,79,80] and provides high-contrast 2D *z*-stack images of skin morphological structures with a subcellular lateral (<1 µm) and axial (<5 µm) resolution, with a screening depth of ≤200 µm, depending on the skin area, excitation wavelength, and regime used [81]. In practice, high-quality images could be acquired down to a depth of 100 µm in the skin. CLSM generally operates in two regimes—reflectance and fluorescence—under one-photon excitation in the broad spectral range of blue to near-infrared wavelengths. The reflectance regime of CLSM is completely non-invasive and provides high-quality images of skin layers sufficient for skin diagnostics [82,83]. The autofluorescence (AF) intensity of the skin is highest when excited with short wavelengths (e.g., with the commonly used blue light) and decreases with increasing excitation wavelength (e.g., it is lowest when excited with red and near-infrared light), and declines significantly from the skin surface with increasing skin depth [84], and is also characterised by strong AF photobleaching [84,85]. The AF emission spectrum of the skin surface does not normally exceed 700 nm [86]. However, in the deep skin, near-infrared-excited AF is detected in the spectral range beyond 1000 nm [87] and is mainly caused by melanin [88]. The increased red- and infrared-excited AF in the SC can be attributed to the presence of oxidation products [8], which may potentially affect penetration studies and should be considered. To visualize skin layers down to the reticular dermis, the intradermal injection of fluorescent dyes with different action spectra (e.g., curcumin, Nile red, and sodium fluorescein; more fluorescent dyes are reviewed in [73]) is required. This procedure is low-invasive with the corresponding ethical limitations for in vivo measurements, for example, on patients.

### 3.2. Fluorescence CLSM (FCLSM)—Skin Penetration Studies

**Figure 5:**
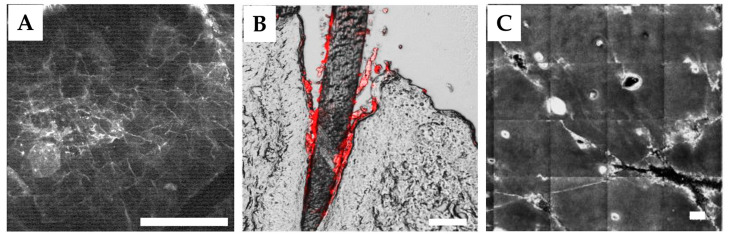
Representative CLSM images (recorded in fluorescence (**A**,**B**) and reflectance (**C**) regimes) of skin penetration. (**A**): Penetration of curcumin-labeled almond oil measured in vivo in human SC (white areas, excitation/emission at 488/590 nm), reprinted with permission from Ref. [92]; (**B**): Penetration of biocompatible hydroxyl ethyl starch nanocapsules measured ex vivo in porcine skin (fluorescence depicted in red, excitation/emission at 555/593 nm), reprinted under CC BY 4.0 license from Ref. [93]; (**C**): Penetration of gold microparticles measured in vivo in human epidermis (white spot areas, reflectance regime at 785 nm), reprinted with permission from Ref. [94]. Scale bar: 100 µm.

The FCLSM is often used in penetration studies where the main requirement is that the investigated substances are inherently fluorescent, or covalently bound with a fluorescent dye [89,90,91]. However, this intervention may affect the penetration kinetics due to the induced changes in the molecular structure of the formulation or target substance. A representative image of the penetration of curcumin-labeled almond oil in skin is shown in Figure 5A. Blue–green excitation wavelengths are usually used [73], because the excited fluorescence intensity is high enough for accurate detection. 

For instance, the excitation at 568 nm has been reported to be preferential for the excitation of skin endogenous AF [89]. The red and near-infrared excitation wavelengths generate only very weak fluorescence signals [8] and are, therefore, mainly used in the reflectance regime of the CLSM. Dual labelling allows the determination of different substances measured sequentially in different excitation/emission fluorescence channels [89,95]. Thus, the depth-dependent accumulation of fluorescent substances, such as polystyrene fluorescein 5-isothiocyanate (FITC)-containing particles (excitation at 488 nm) [89], fluorescent polymeric nanoparticles (excitation at 488 nm) [96], rhodamine B base loaded with a phospholipid surfactant conjugated with a green fluorophore (excitation/emission at 493/517 and 577/603 nm) [95], Nile red in different vehicles (excitation/emission at 514/550 nm) [97], fluorescent model substances of increasing lipophilicity (excitation/emission at 488/514 and 564/574 nm) [98], liposomal formulation containing hydrophilic carboxyfluorescein (excitation/emission at 470/520 nm) [99], Cy7-loaded calcium carbonate particles (excitation/emission at 405/(420–490) nm) [100], quantum dot fluorescent nanoparticles of different modifications (excitation/emission at 488/565 nm) [101], nanodiamonds (excitation/emission at 532/(650–720) nm) [102], gold nanoparticles coated with a mixture of hydroxyl- and carboxyl-terminated thiolates (excitation at 485 and 543 nm) [103], the infrared-triggered release of FITC gold nanoparticle-doped bovine serum albumin (excitation/emission at 488/580 nm) [60], FITC-silica particles (excitation/emission at 488/(500–550) nm) [104], and curcumin [105] can be easily visualized in the skin, and the penetration depth and pathways can be determined. For example, using the FCLSM, the release of the fluorescent cytostatic chemotherapeutic drug doxorubicine with sweat on the skin surface and its subsequent penetration and accumulation in the SC of glabrous skin can be successfully determined [106,107]. Based on these results and the doxorubicine-induced decrease in the concentration of antioxidants in the SC, it was hypothesized that the probable cause for the development of hand–foot syndrome is the damaging interaction of doxorubicine and/or its metabolites with the SC compartments, leading to barrier disruption [108]. The accumulation, penetration pathways, and removal efficacy of sodium-fluorescein-labeled soot contaminant particles from skin were demonstrated by FCLSM ex vivo [109]. The in vivo and ex vivo imaging of hair-follicle-containing skin areas do not provide fully depth-resolved images, which is due to the limitation of the screening depth and the “shadows” caused by the hair shaft due to its curved position in the skin, so that deep penetration into the hair root and dermal papillae regions are not detectable [110]; the investigations on vellus instead of terminal hair follicles could be recommended. Follicular penetration is easier to analyze on skin biopsy cryo-sections ex vivo, where the entire hair follicle is visible [73,93], which is shown in Figure 5B. Here, the quantitative analysis is hardly possible [111].

CLSM combined with FLIM requires short-pulse laser excitation. This technique has been used ex vivo to determine the penetration depth of dye-tagged dendritic core multishell nanotransporters and Nile red into the normal and barrier-disrupted skin [112], and in vitro to demonstrate the loading/release of doxorubicine within/from the gold nanoparticle carriers [113]. Perfect agreement between the results of CLSM-FLIM and 2PT-FLIM has been demonstrated [113]. Although CLSM is popular in penetration studies, this method provides semi-quantitative information about penetrants and has numerous limitations. 

### 3.3. Reflectance CLSM (RCLSM)—Skin Penetration Studies

The RCLSM can be used for the determination of the penetration of exemplary particulate formulations, characterized by an enhanced reflectance in the certain optical range [94,114]. Preferably, the excitation at 785 nm is used, as it is in the “skin optical transparent window-I” (Figure 3) and, thus, penetrates deep into the skin. An example of the visualization of highly reflective gold microparticles in skin in vivo using RCLSM imaging is shown in Figure 5C. Formulations without particles do not have specific reflectance properties and are, therefore, not detectable. Consequently, the RCLSM is rarely used in skin penetration studies.

### 3.4. CLSM—Advantages, Limitations, and Applied Substance Requirements 

The main advantages of in vivo reflectance/fluorescence CLSM in skin penetration research are its non-invasiveness, fast image acquisition, high-quality morphological imaging with a subcellular resolution, and large skin-screening areas—up to 8 mm × 8 mm. The price of CLSM is lower compared to other optical microscopy methods, which also serves as an advantage. The limitations include the maximal screening depth of ≈100–150 µm (less in the presence of hair follicles), and the ability to examine only fluorescently active formulations (for FCLSM) or highly reflective particles (for RCLSM).

For successful CLSM studies, the formulation should be inherently fluorescent or covalently bound with a fluorescent dye so that its fluorescence intensity exceeds that of skin fluorescence (for FCLSM), or it should contain highly reflective particles (for RCLSM).

## 4. Multi-Photon Tomography (MPT)

MPT is a 3D optical imaging method based on the detection of fluorescence and harmonic signals excited by multiple photons. At high photon flux, two or three photons can be absorbed simultaneously and the sum energy is sufficient to put the molecule in the excited state; then, the emitted two- or three-photon-excited fluorescence is measured (Figure 4A—non-linear microscopy—shows the process schematically). Pulsed fs lasers are usually used for this purpose. Confocality is achieved automatically and without a pinhole, as the probability of two- or three-photon absorption is extremely low and is realized only in the focal plane from a volume of the order of a femtoliter (Figure 4B). The working principle of the MPT is described in detail elsewhere [75,115,116]. 

### 4.1. Two-Photon Tomography (2PT) in Skin Morphology Imaging

Two-photon tomography is frequently applied in in vivo dermatological research [116,117,118,119], mainly uses excitation in the range of 760–820 nm (“skin optical transparent window-I” (Figure 3)), and provides high-quality-resolved images of skin structures from the surface down to ≈150–200 µm with a subcellular lateral (<0.6 µm) and axial (<2 µm) resolution. The standard 2PT operates with two channels—autofluorescence (AF) and second-harmonic generation (SHG)—which provide information on the distribution of AF-generating fluorophores representing the main morphological features of the skin (cells, melanin, and elastin) [116], and SHG shows the distribution of non-centrosymmetric collagen type I in the dermis [116] and recently discovered crystallized urea dendriform structures in the SC of glabrous skin [77]. An extended version of the 2PT, additionally combined with spectral imaging of the AF [120], FLIM [116], CARS [121], and/or RCM [122] channels, considerably expand the possibilities for molecular imaging and practical applications. 

The spectral imaging of AF offers the advantage of seeing the distribution of emitted AF photons as a function of wavelength, which is important information for the choice of the appropriate AF transmission filters [123] and for the determination of the penetration of topically applied fluoresce substances with AF spectra different from the skin spectra [120]. The 2PT-FLIM technique is a time-resolved technique that enables the detection of fluorescence lifetime decay curves, determining fast (>0.2 ns) and slow (<2.4 ns) lifetime components, which are described mainly by free and protein-bound NAD(P)H, respectively, with increased free NAD(P)H indicating reduced metabolic activity [124,125]. Fluorescence lifetimes are dependent on the chemical composition of the target fluorophores and their interaction with the surroundings [126], and vary for different skin constituents [119]. Thus, based on the individual lifetime characteristics, it was possible to image melanin [127], collagen type III [128], mast cells [129], and macrophages [130] in skin in vivo and ex vivo, to determine the metabolic changes during keratinocyte proliferation in vitro [131] and to differentiate and diagnose skin cancer ex vivo [132] using 2PT-FLIM. The 2PT-CARS technique is able to visualize the distribution of lipids and water in the skin in vivo and ex vivo [133]. The combination of 2PT with the reflectance regime provides a fast overview provided by RCLSM [122,134] with the possibility of detailed multimodal 2PT imaging of the area of interest [135]. The operating principle of 2PT-(AF, FLIM, RCM, SHG, and CARS) is described elsewhere [76,121,122].

### 4.2. 2PT-AF—Skin Penetration Studies

**Figure 6:**
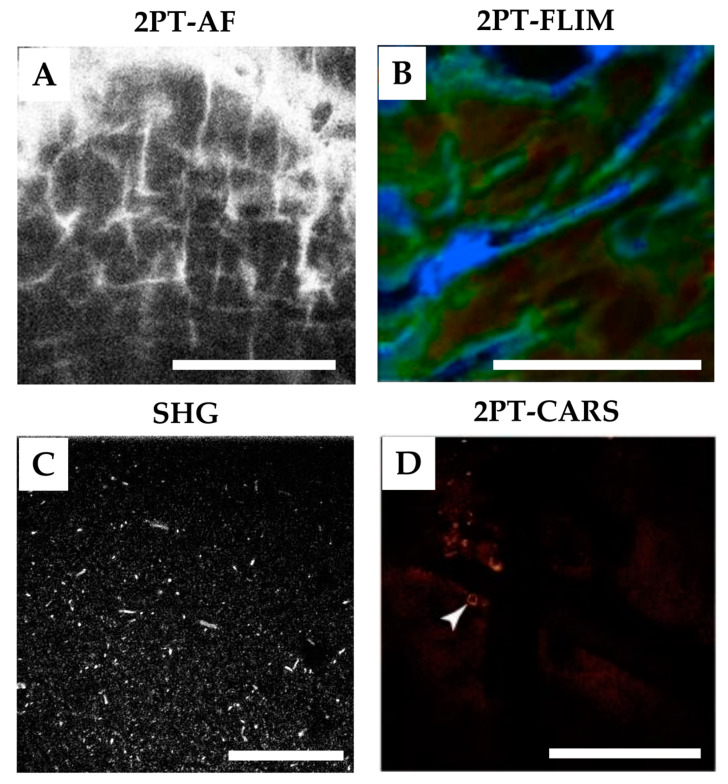
Representative 2PT-AF (**A**), 2PT-FLIM (**B**), SHG (**C**), and 2PT-CARS (**D**) images of skin penetration. (**A**): Penetration of sunscreen labeled with 0.1% fluorescein into human SC in vivo (homogeneously distributed white areas, 2PT-AF excited at 760 nm), adopted with permission from Ref. [143]; (**B**): Penetration of Nile red into porcine SC ex vivo (green–blue areas represent the Nile red (mean AF lifetime 2000–2500 ps), 2PT-AF excited at 760 nm), adopted under CC BY 4.0 license from Ref. [145]; (**C**): Penetration of zinc oxide nanoparticles (0.008%) into human SC in vivo (homogeneously distributed white particles, SHG excited at 760 nm), reprinted with permission from Ref. [146]; (**D**): Penetration of tailorable nanoemulsions packed with glycerol into human epidermis ex vivo (white arrow shows large aggregate, 2PT-CARS excited at 1040 nm for “Stokes” and 803 nm for “pump” beams), adopted under CC BY-NC-ND 4.0 license from Ref. [114]. Scale bar: 50 µm.

The 2PT-AF (intensity and spectral channels) technique is a valuable non-invasive method to study skin penetration. If the applied substance exhibits intense two-photon-excited AF whose intensity exceeds skin AF and/or whose AF emission spectrum differs from that of the skin constituents, this can be visualized in the skin [120,136]. For instance, under two-photon excitation at 780 nm, the fluorescence emission maxima for the hydrophobic rhodamine B hexyl ester and hydrophilic sulforhodamine B fluorescent dyes at 578 and 586 nm, respectively, overlap minimally with skin AF. This allows their visualization and quantification in the skin, as well as an estimation of the effect of oleic acid as a penetration enhancer [137], and the visualization of the intracellular penetration pathway [138]. Skin penetration was determined for topically applied highly fluorescent 6-carboxyfluorescein in a core droplet of the tailorable nano-emulsions [114], fluorescently labelled dextran in combination with topical enhancers [139], gold nanoparticle dispersion [140], zinc oxide nanoparticles [141], nanobeads [142], FITC dextran, Texas Red [120], FITC-labeled PLGA nanoparticles [136], and sunscreen labeled with fluorescein dye [143]. A representative image of the latter is shown in Figure 6A. The distribution and deposition in the SC, furrows, and hair follicles could be clearly visualized. In addition, the accumulation of the highly fluorescent chemotherapeutic drug doxorubicine exclusively within the cytoplasm in the perinuclear area has been demonstrated in vitro in chemo-sensitive living cells and their chemo-resistant variants using 2PT-AF [144], suggesting the possibility of visualizing doxorubicine in the skin of chemotherapy patients in vivo (inside–outside penetration). The major limitation of 2PT-AF (intensity and spectral channels) is the presence of skin intrinsic AF intensity and broad AF emission spectrum, which make this method not sensitive enough in most practical cases.

### 4.3. 2PT-FLIM—Skin Penetration Studies

The 2PT-FLIM technique is a pseudo-chemical analysis, which significantly improves and extends the capabilities of 2PT-AF for skin penetration studies by providing additional information on the fluorescence lifetime parameters of the target substances. The applied substances, which have their own two-photon-excited AF, usually characterized by the combination of fast and slow AF lifetimes (that are normally used as parameters for bi-exponential fitting [76]) that are different from the AF lifetime values of the endogenous skin constituents, can potentially be evaluated in the skin [147]. The 2PT-FLIM technique was used to determine the skin penetration depth and pathways for topically applied poly-N-vinylpirrolidone-coated silver nanoparticles [148], uncoated silver nanoparticles [149], nanobeads [142], nanodiamonds [102], minocycline and tazarotene—used for the treatment of acne vulgaris [150,151], anti-inflammatory compound GSK2894512—a drug used for the treatment of atopic dermatitis and psoriasis [152], ethinyl estradiol—used in hormonal therapy [153], bovine serum albumin and hyaluronic acid [154], sodium fluorescein [145,147], Nile red [112,145], 5-carboxyfluorescein-labelled liposomes [147], coated and uncoated zinc oxide nanoparticles [149,155,156], gold nanoparticles [157], and dendritic core multishell nanotransporters [112]. It was recently shown that the penetration of non-fluorescent propylene glycol can also be determined indirectly in skin based on the pH-dependent increase in the AF lifetime of SC components [145]. A representative image of the penetration of Nile red into porcine SC ex vivo is shown in Figure 6B. The 2PT-FLIM technique is also applicable for the determination of inside–outside penetration, which was recently demonstrated in vivo for carbon black tattoo ink particles diffusing from the dermis into the epidermis in old tattoos [158]. The results demonstrate the possibility of identifying the penetration profiles of multicomponent substances based on the differences in their fluorescence lifetime parameters [151]. The 2PT-FLIM technique can also be successfully used for the visualization of drugs, such as proretinal and retinal nanoparticles, delivered with microneedles [159]. To distinguish between the endogenous and exogenous skin FLIM data, a phasor approach, where the time signal is transformed into a pair of phasor co-ordinates representing the sine and cosine components of a Fourier transform, is highly advantageous [160]. Using a phasor plot, it is possible to visualize many fluorophores simultaneously according to their temporal characteristics [76]. 

### 4.4. SHG—Skin Penetration Studies

SHG does not require electronic excitation of the molecules (Figure 4A—non-linear microscopy—shows the process schematically) and is used to study the penetration of non-centrosymmetric SHG-active substances in the skin, such as zinc oxide nanoparticles, where the intercellular and follicular penetration pathways could be recognized [146]. No zinc oxide nanoparticles were found in viable epidermis [146], suggesting that there is no cellular toxicity induced and it can be safely used in sunscreens [161]. Chemical enhancers such as ethanol, oleic acid, and oleic acid–ethanol, however, facilitate the transdermal delivery of zinc oxide nanoparticles, which is due to the increase in lipid fluidity and/or the extraction of lipids from the SC [162]. This method is very sensitive for screening the SHG-active substances in the SHG-free epidermis. One limitation is glabrous skin whose SC may contain SHG-active crystallized urea dendriform structures [77], which cannot be separated from the applied formulation. A representative image of the penetration of zinc oxide nanoparticles into the SC of human forearm skin in vivo is shown in Figure 6C.

### 4.5. 2PT-CARS—Skin Penetration Studies

With CARS, the vibrational signatures of the molecules can be determined. Three laser beams (ps or fs pulses) are required to excite CARS: a “pump” (frequency ω_p_), a “Stokes” (frequency ω_S_), and a “probe” beam (frequency ω_pr_) [163]. However, to simplify the setup, the “probe” and “pump” beams are often provided by one laser and have the same frequencies [123]. The interaction with the sample leads to the generation of a coherent optical signal at the anti-Stokes frequency ω_CARS_ = ω_pr_ + ω_p_ − ω_S_ = 2ω_p_ − ω_S_, which is strongly enhanced when the energy difference between ω_p_ and ω_S_ matches the energy of molecular vibration Ω = ω_p_ − ω_S_. The excitation of the CARS signal does not require electronic excitation of the molecules (Figure 4A—non-linear microscopy—shows the process schematically). The working principle of the 2PT-CARS is described in detail elsewhere [75,121,123]. 

The 2PT-CARS technique allows the multimodal imaging (imaging speed can be <1 s/image [164]) of human skin ex vivo and in vivo with a subcellular resolution [72,121,133,165,166], but is not commonly used in skin penetration studies. It has been shown that the use of 2PT-CARS can visualize the penetration of airborne carbonaceous particulate materials (2693 and 2840 cm^−1^) [167], retinol (1594 cm^−1^) [168], and elongated silica microparticles combined with tailorable nanoemulsions packed with glycerol (2845 cm^−1^) [114] in skin ex vivo. A representative image of the latter is shown in Figure 6D. Here, the 2PT-CARS is excited at 1040 nm for the “Stokes” and 803 nm for the “pump” beams (“skin optical transparent window-II and -I”, respectively (Figure 3)). The 2PT-CARS technique can also be successfully used for the visualization of drugs, such as betamethasone dipropionate (1750 cm^−1^), delivered with microneedles [169]. In vivo studies examined the penetration of omega-3-oil (2845 cm^−1^) [133] and mineral oil (2845 cm^−1^) [170] into the epidermis, but there was no effective separation between the applied oil and SC lipids, which appears to be a major limitation of this method in penetration studies. Although CARS bands are normally not overlapped with the fluorescence background [123], the strong overlap of lipid- and protein-related Raman bands is also a critical factor in evaluating their separate contributions in the HWN spectral region [171]. Successful quantitative determination is possible for substances whose Raman spectra do not overlap with the skin spectrum, such as deuterated glycerol [172]. 

### 4.6. Three-Photon Tomography (3PT) in Skin Imaging and Penetration Studies

In skin analysis, 3PT mainly uses longer excitation wavelengths (≈1200–2200 nm with an optimum at ≈1300 nm, ≈1700 nm [71,173], or 2200 nm [174]) corresponding to “skin optical transparent window-II, -III, and IV” (Figure 3), which are known for the deeper penetration of light into the skin compared to 2PT, where the typical excitation wavelengths are in the spectral region of 710–920 nm [123]—“skin optical transparent window-I” (Figure 3). The higher imaging depth of 3PT compared to 2PT is due to the lower absorption (mainly by water) and the lower scattering of both excitation and emission light (Figure 3). It has been reported that the possible photodamage with three-photon excitation is lower than with two-photon and even one-photon excitation, which is due to the lower absorption by water and lower heating [175]. The imaging speed can be <1 s/image [164]. The endogenous three-photon-excited AF of skin is weak (3PT-AF images are background-free); therefore, the high-contrast exogenous three-photon-excited fluorescent dyes, such as green and red fluorescent proteins [71], iridium (III) complexes [176], moxifloxacin [177], or nanoparticulate materials [178] are required to provide bioimaging. Third-harmonic generation (THG) does not require electronic excitation of the molecules (Figure 4A—non-linear microscopy—shows the process schematically) and is sensitive to local differences in the third-order nonlinear susceptibility, refractive index, and dispersion, and is particularly generated by water–lipid or water–protein scaffold interfaces, as well as lipid bodies, fat cells, nerve fibers, membranes, intracellular vesicles [179], and blood capillaries [164]—it does not require exogenous dyes, i.e., is non-invasive. 

Studies on skin penetration with 3PT are lacking in the literature. However, 3PT has a strong potential to visualize particulate substances in a similar way as shown for tumor-associated microparticles and aggregated intracellular vesicles in skin in vivo [179], gold nanorods in the skin and brain in vivo and ex vivo [180], the deposition of intravenously injected gold–silver nanocages in mouse liver ex vivo [181], or lipid droplets in mouse liver ex vivo [182]. The latter may mimic oil-in-water/water-in-oil pharmaceutical formulations. The combination with FLIM should greatly broaden the applicability of 3PT in future skin penetration research. 

### 4.7. MPT—Advantages, Limitations, and Applied Substance Requirements

The major advances of 2PT over one-photon CLSM imaging in in vivo skin penetration research are its non-invasiveness, high-quality morphological imaging with a subcellular resolution due to signal generation in a small volume (Figure 4B), and increased imaging depth (Figure 3) to ≈150–200 µm (less in the presence of hair follicles). Since two-photon absorption occurs in a subfemtoliter volume for a very short time (fs), the skin undergoes negligible photobleaching [149] and phototoxicity [160]. The main drawbacks are the high cost of the 2PT device, the not-very-fast image acquisition (usually 3–12 s/image), and the small skin-screening areas (max. ≈ 300 µm × 300 µm) that make it difficult to find target areas quickly. The 3PT technique has the prospect of being used in skin penetration research.

Substances studied with 2PT-AF in skin should exhibit intense two-photon-excited AF whose intensity exceeds that of skin and/or whose AF emission spectrum differs from that of skin (for 2PT-AF and 2PT-FLIM). This can potentially be realized by using new materials excited in “skin optical transparent window-II” and emitted two-photon-excited AF in “skin optical transparent window-I” (Figure 3), where the intrinsic 2PT-AF intensity of the skin is minimal, such as monomeric and dimeric di-styryl-BODIPY dyes [183]. For the SHG analysis, target substances should be SHG-active. For the 2PT-CARS analysis, the most important requirement for the target substances is the presence of a Raman band that does not overlap with that of skin, preferably in the ≈1700–2820 cm^−1^ spectral region. The overlap of the Raman bands of the substance and skin reduces the detection sensitivity. Potential limitations of using 3PT in skin penetration research include studying only those substances that exhibit three-photon-excited fluorescence and/or THG-active substances.

## 5. Confocal Raman Micro-Spectroscopy (CRM)

CRM is a spectroscopic method with chemical sensitivity based on the detection of inelastically scattered photons containing information about the vibrational modes of molecules when excited by a monochromatic continuous-wave laser (spontaneous Raman scattering). Stokes scattering is only measured with CRM due to its higher intensity compared to anti-Stokes scattering (Figure 4A—linear microscopy—shows the process schematically). The movement of the objective allows the spectra to be recorded at different positions. Depth scanning is performed by moving the focus within the sample. Since the sample is also excited outside the focal plane (Figure 4B), confocality is achieved by using an optical fiber whose core (100 µm diameter) acts as a confocal pinhole to block photons from outside the focal plane [184]. Thus, only photons excited in the focal plane produce a final Raman spectrum. The working principle of the CRM is described in detail elsewhere [184,185,186]. 

### 5.1. CRM in Chemical Skin Research

Conventional CRM uses spontaneous Raman scattering for the chemical determination and characterization of skin (preferentially SC) constituents’ [184,187] and physiological parameters’ depth profiles [188], in the numerous in vivo and ex vivo skin penetration studies [31,37,189,190,191,192,193,194,195,196,197,198,199,200,201,202] and the study of the influence of cosmetics on the physiological parameters of the SC in vivo [203,204,205]—a challenging task in skin research [186]. The unique features of the CRM are its non-invasiveness for skin constituents and target substances, the lack of need for fluorescent or other markers, and the ability to perform chemical analyses quantitatively using various calibration approaches [206,207,208,209,210,211,212,213,214]. In most cases, red and near-infrared excitation wavelengths are used, which have low absorption and scattering in the skin [215] (“skin optical transparent window-I” (Figure 3)) and, therefore, penetrate deep and excite the Raman spectra of skin constituents and applied substances with a very low fluorescence intensity, making the Raman bands easy to determine. The CRM suitable for in vivo skin measurements is manufactured exclusively by RiverD International B.V. (Rotterdam, The Netherlands). The CRM uses two lasers with wavelengths of 785 nm and 671 nm to excite the Raman spectra in the fingerprint (FP: 400–2200 cm^−1^) and high wavenumber (HWN: 2500–4000 cm^−1^) regions, respectively, at a laser power on the skin surface of ≤20 mW. The spectral resolution of the CRM is 2 cm^−1^ and the axial resolution is <5 µm. Normally, the CRM is capable of acquiring high-quality Raman spectra down to a depth of ≈50 µm in the skin—i.e., the entire epidermis and papillary dermis could be analyzed on forearm skin [88,187,216,217]—however, the analyses are mainly limited to the SC.

**Figure 7:**
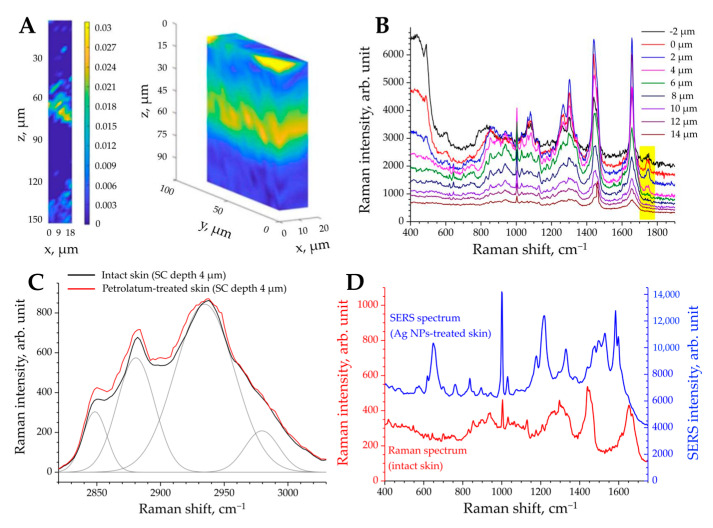
Representative CRM images and Raman spectra of treated skin. (**A**): 2D (left) and 3D (right) penetration depth profiles of hyaluronic acid (10 kDa) into porcine skin ex vivo, adopted under CC BY-NC-ND 4.0 license from Ref. [218]; (**B**): 1D z-stack point measurement (depth profile) Raman spectra of SC of jojoba oil-treated skin (yellow highlighted area indicates presence of jojoba oil—specific band at 1740 cm^−1^); (**C**): HWN Raman spectra of SC (depth 4 µm) of intact (black) and petrolatum-treated (red) skin (the spectrum of intact skin is decomposed using four lines described with Gaussian functions—shown in grey); (**D**): The typical SERS spectrum of skin treated with silver nanoparticles (blue, depth 4 µm, SC) compared to the spontaneous Raman spectrum of intact porcine skin ex vivo (red, depth 4 µm, SC), adopted under CC BY 2.0 license from Ref. [223].

In contrast to the CLSM and 2PT methods, where a fast acquisition time is required to record 2D images (usually, seconds), high-quality skin imaging using spontaneous Raman scattering CRM (the 2D *z*-stack images of skin sections) is only possible ex vivo, which has been shown in numerous studies [186,191,199,202,218,219]. This is due to the long acquisition time required to accumulate enough spontaneously scattered Raman photons to create an image (usually, hours), which includes signal acquisition for each measurement point (usually, 1–10 s/point) and scanning the skin section to create a 2D or 3D image (usually with >500 points). The need for an additional change of the excitation wavelength or grating position (usually required for the acquisition of Raman spectra in the FP and HWN regions) increases the total acquisition time considerably (usually by hours) [219]. The representative 2D and 3D images of hyaluronic acid penetration in skin ex vivo are shown in Figure 7A. The long acquisition time and inability to quickly create 2D/3D images are a major limitation for in vivo skin imaging using spontaneous Raman scattering CRM, which can be overcome by the non-linear coherent anti-Stokes Raman scattering (CARS), stimulated Raman scattering (SRS) microscopy, or surface-enhanced Raman scattering (SERS) methods, discussed below. Thus, CRM in vivo provides the 1D *z*-stack point measurements (depth profiles), and the typical Raman spectra are shown in Figure 7B. To precisely guide the Raman point measurements in vivo, the CRM could be combined with the RCLSM [220], OCT [221], or line-field confocal OCT [222] imaging modalities that rapidly visualize skin morphology at a subcellular resolution and allow the operator to assess a region of interest for Raman point measurements. 

The most important prerequisite for the determination of the penetration profile of the applied formulation (target substance) in the skin is the presence of differences between the Raman spectra of the untreated and formulation-treated skin, whose spectra always overlap completely or partially. Pre-processing routines for Raman spectra are always required prior to meaningful analyses, and include following procedures: wavenumber/intensity calibration prior to measurements; cosmic spike correction; spectra smoothing; noise reduction (e.g., using principal component analysis); and fluorescence background subtraction (mainly using higher-order polynomial and/or linear functions) [171,184,224,225]. The following six methods are known to be used in Raman spectra analysis to determine penetration profiles of target substances into skin. 

### 5.2. “Tracking Specific Raman Band” Method—Skin Penetration Studies

The “tracking specific Raman band” method accounts for the largest difference between the untreated and formulation-treated skin in a narrow spectral region (typically <100 cm^−1^), and is limited to a formulation-specific Raman band. The highest difference is observed when no overlap occurs in the Raman spectra of the skin and formulation, i.e., when the formulation-specific Raman band is in the spectral region where no skin band is present (≈1700–2820 cm^−1^), or the bands are weak. To compensate for depth-dependent signal attenuation due to the absorption and scattering of light in the skin, normalization on proteins (keratin) is always used [184,192,226,227,228,229]. This method has been successfully applied to study the penetration of vegetable oils (almond oil, soybean oil, olive oil, jojoba oil, and avocado oil) into the skin by tracking the intensity of the specific Raman band at 1740 cm^−1^, corresponding to the vibration of the carbonyl group C=O in vegetable oils, which is not present in intact skin [192], and of procaine (Raman band at 1612 cm^−1^, corresponding to ν(NH) δ(C-C) vibration) [226,228]. The typical depth-resolved FP Raman spectra of human SC of jojoba-oil-treated skin (the yellow-marked spectral region at ≈1740 cm^−1^ shows an oil-specific band) are represented in Figure 7B. The sensitivity of this method can be increased by applying, for instance, a multivariate chemometric analysis, such as a combination of principal component analysis (PCA) and linear discriminant analysis (LDA), which will maximize the difference between two groups. This was demonstrated in determining the penetration depth profiles of caffeine (Raman band at 555 cm^−1^, corresponding to the C=O–N deformation mode of caffeine) and propylene glycol (Raman band at 840 cm^−1^, corresponding to the (C–O)H stretching mode of propylene glycol) [230] in skin. The maximum penetration depth can be determined comparing untreated and formulation-treated skin at a certain skin depth. The enhancement of the intensity of a specific spontaneous Raman scattering band is possible and is referred to as stimulated Raman scattering (SRS), described below in the section “SRS microscopy”. 

The distinct advantage of the “Tracking specific Raman band” method is the ability to visually monitor the presence of the formulation (target substance) at a certain depth in the SC during the measurement. The limitation is the inability to measure the formulations or substances without having a specific Raman band.

### 5.3. “Non-Restricted Multiple Least Squares Fit” Method—Skin Penetration Studies

The “non-restricted multiple least squares fit (NMF)” method was proposed by Caspers et al. [184] and is implemented in the “Skin Tools” software from RiverD International B.V. (Rotterdam, The Netherlands). The NMF method is based on mimicking the FP Raman spectra of the SC by a set of known model Raman spectra of SC constituents, including keratin, ceramide 3, cholesterol, urea, lactate, *trans*-urocanic acid at pH4 and pH8, natural moisturizing factor, and water, using the least-squares fitting procedure with minimization of the residual error. As a result, the fitting amplitudes represent the semi-quantitative concentrations of the corresponding SC constituents. After adding the FP Raman spectrum of the formulation, the NMF method calculates the depth profile of the formulation in the SC semi-quantitatively or quantitatively [207]. The main advantage of the NMF method is the analysis of the entire FP region, which includes all possible contribution of the applied formulation in the SC. Among the disadvantages are the frequently occurring artifacts in the penetration depth profiles of an unknown nature in deep areas of the SC, as well as the inability to account for the changes in the model spectra at different SC depths (caused by interaction with the surrounding environment). To date, the NMF method has been widely used worldwide and serves as the “gold standard” in determining the penetration depth profiles of topically applied formulations into the SC in vivo or ex vivo, which was shown for dimethyl sulfoxide (DMSO) [189], caffeine and quinidine [36], hydroxyethyl cellulose and hydroxypropyl methylcellulose [65], mycosporine-like amino acids and gadusol photoprotectants [231], cosmetic oils (that have not permeated the SC) [192], essential oils (that have permeated the SC) [232], urea-containing moisturizing creams [233], soap [234], silver nanoparticles [148], nano-sized lipid particles and base cream [35], niacinamide [235], nicotine [236], and carotenoids [237]. 

### 5.4. “Partial Least Squares Regression” Method—Skin Penetration Studies

“Partial least squares regression (PLSR)” is a supervised multivariate analysis method that correlates the changes in the positions, intensities, and shapes of Raman bands, and is used to relate two groups of variables (untreated and formulation-treated skin) by quantifying the difference between them across the selected spectral range [213,238]. PLSR is not able to separate the skin constituents, but is well-suited to quantify the presence of the formulation (difference between untreated and formulation-treated skin) at all measured skin depths. The PLSR method has been used to determine the penetration of piperonyl esters [213], resorcinol [239], and curcumin-loaded alginate nanocarriers [240] into the skin, and to determine the changes in SC lipids induced by dietary fat intake [241].

### 5.5. “Gaussian-Function-Based Decomposition” Method—Skin Penetration Studies

The “Gaussian-function-based decomposition” method was introduced by Choe et al. [242] and is based on the decomposition of the HWN Raman spectrum of the SC in the spectral region of 2820–3030 cm^−1^ using lines described with Gaussian functions (Figure 7C). This spectral region represents the overlap of protein- and lipid-related broad Raman bands and will always be affected in formulation-treated skin since all types of topical formulations contain lipids [242,243]. The penetration depth of oils into the SC determined by this method was comparable to the results obtained by the “gold standard” NMF method [192]. The main disadvantage of the “Gaussian-function-based decomposition” method can be related to the technical difficulties in programming the decomposition and adjusting the parameters of the Gaussian functions (variation of maximum position and width) to the fitting criteria. For depth profiling, normalization on the intensity of the protein band is required.

### 5.6. “Non-Negative Matrix Factorization” Method—Skin Penetration Studies

The “non-negative matrix factorization (NNMF)” method was recently introduced by Yakimov et al. [187] and is based on the multivariate chemometric analysis, where the depth-resolved FP Raman spectra of the skin are factorized with 8–16 matrices (individual components) that do not contain negative elements. In the formulation-treated skin, these individual components extracted by NNMF match well with the known Raman spectra of the skin constituents and the applied formulation without a priori assignment and knowledge of the Raman spectra of the skin constituents and the applied formulation. The results show that at least two molecular constituents—carotenoids and melanin—are present in the SC and are missing in the spectra library of the “gold standard” NMF method [187]. The main advantage of the NNMF method is the automatic factorization of endogenous and exogenous skin constituents without the need for their Raman spectra or other additional information. Meanwhile, the NNMF method does not require any normalization (division on protein-related Raman band intensity [184,227]), which extends its applicability from the SC to the entire epidermis and dermis (limited only to the screening depth of the CRM in skin). 

### 5.7. “Tailored Multivariate Curve Resolution–Alternating Least Squares” Method—Skin Penetration Studies

The “tailored multivariate curve resolution–alternating least squares (tMCR-ALS)” chemometric method was recently introduced by Choe et al. [209,244] and is applied in quantitative skin penetration studies using the analysis of the HWN [209] and FP [245] spectral regions. The tMCR-ALS method handles the spectral region of the input skin spectra and optimizes the convergence process to minimize the difference between the modelled and real spectra of the endogenous and exogenous skin constituents, and uses the adapted source code “MCR-ALS GUI 2.0” [246], which has been used in skin constituent analysis [247] and penetration studies [218,248,249]. In tMCR-ALS, the skin Raman spectra are decomposed into loadings and scores matrices using an iterative alternating least squares method, where loadings refer to the individual skin constituents and scores represent their corresponding concentrations [219,250]. 

The main advantage of the tMCR-ALS method, similar to the NNMF method, is the broad spectral region analysis instead of a single band analysis, and that the depth profiles of the endogenous skin constituents and the topically applied formulation can be determined simultaneously without the need for a normalization procedure, even if their spectra are unknown. Thus, the resulting profiles have no normalization-related artifacts and other limitations for the analysis [227,251,252]. Moreover, the applied formulation does not affect the determination of the profiles of the skin constituents, whose Raman spectra always overlap. The stronger the spectra collinearity (similarity), the lower the method sensitivity.

### 5.8. CRM—Advantages, Limitations, and Applied Substance Requirements

The main benefits of CRM in in vivo skin penetration research are its non-invasiveness, no need for labelling, and the ability to provide high-quality chemical information about the SC constituents and the formulation (its target substances) used. The ability to identify the formulation stability in the skin (formation of metabolites of the formulation) is also an advantage of CRM, which is a challenging future task. The main weaknesses are the high cost of the CRM device; the need for two excitation lasers or grating movement to acquire the FP and HWN regions; the not-very-fast acquisition time (1–10 s/point), and, as a result, the inability to acquire 2D/3D images in vivo (long acquisition time); the relatively complicated analytical procedures; and the limited screening depth.

The main requirement for the substances used is the presence of differences in their Raman spectra from that of the skin (the more spectral differences there are, the higher the sensitivity is in detecting substances in the skin)—on this basis, one of the methods of Raman spectra analysis discussed above can be chosen. The presence of substance-related intense fluorescence in the Raman spectrum is not critical, as this information can be used additionally to visualize the substance in the skin depths. 

## 6. Surface-Enhanced Raman Scattering (SERS) Microscopy

SERS provides multiple enhancements of Raman signal intensity due to the localized surface plasmon resonance generated by noble metal or nitride-based nanostructures [253], the sensitivity of which depends on the material and surface design of the nanostructures [254,255]. Thus, SERS represents a unique technique that allows the detection of signals originating from single nanoparticles [256] that makes the control of delivery and penetration measurements very sensitive, which cannot be achieved by other methods. Here, the “skin optical transparent window-I” is mainly used for excitation, but the excitation in the visible spectral region could also be used, albeit with a much lower penetration depth into the skin (Figure 3). The microscope used for recording the SERS spectra is the same CRM used for recording the spontaneous Raman spectra, described in the section “CRM”.

### 6.1. SERS—Skin Penetration Studies

The penetration of SERS-active silver [148,223,257] and gold [258,259] nanoparticles has been successfully studied by tracking the appearance of the SERS signal at a certain skin depth. The release of coated silver nanoparticles within the skin has also been demonstrated by the appearance of the SERS spectra [148,223], whose shape and intensity are very different from the spontaneous Raman scattering spectrum of intact skin (Figure 7D). The rapid non-invasive determination of the presence of a drug in sweat was performed using plasmonic silver nanowires, which, after contact with sweat components, generate an SERS signal [260]. The inside–outside transdermal penetration of cortisol with sweat can also be detected using functionalized silver triangle nanoplates [261]. SERS-tag-labeled detection microneedles can be used to detect the drug release, diffusion behavior, and treatment effect in the skin by analyzing the interstitial fluid [262,263,264]. 

An advancement of SERS is tip-enhanced Raman spectroscopy (TERS), which allows the lateral resolution limit of CRM to be overcome down to 2 nm [186], and has been used in skin penetration studies. The high-resolution TERS images were recorded for intact invasome vesicles that penetrated into the SC (in vivo, low-invasive tape strip analysis) [265]. 

### 6.2. SERS—Advantages, Limitations, and Applied Substance Requirements

The main advantages of SERS in in vivo skin penetration research are the very high chemical sensitivity based on the signal acquisition from a single SERS-active nanostructure and the ease of spectral analysis. The restrictions are the high cost of the equipment and the need for special permits for in vivo measurements.

In order to detect the substance-related SERS spectrum in the skin, the applied substances should be SERS-active; i.e., they should contain encapsulated or uncovered noble metal or nitride-based nanostructures.

## 7. Stimulated Raman Scattering (SRS) Microscopy

SRS microscopy is a label-free, non-destructive tool for 2D/3D spectroscopic chemical imaging [266,267,268,269,270], which is a resonance enhancement of the spontaneously excited Raman scattering signals used in conventional CRM. To create a coherent and stimulated condition, two “pump” (frequency ω_p_) and “Stokes” (frequency ω_S_) laser beams (ps or fs pulses) need to be focused on the skin sample. The frequency of the “Stokes” laser beam is selected so that the difference from the “pump” laser beam Ω = ω_p_ − ω_S_ resonantly matches a Raman band frequency of the target molecule (water, lipids, proteins, and substance of interest—for the penetration study). Excitation of the SRS signal does not require the electronic excitation of the molecules (Figure 4A—non-linear microscopy—shows the process schematically). The imaging speed can be <1 s/image [164], which is an important advantage over spontaneous CRM. The sensitivity of SRS is significantly higher than that of spontaneous CRM, which is achieved by implementing high-frequency phase-sensitive detection, and the signal is background-free with a high chemical contrast [268]. The working principle of the SRS is described in detail elsewhere [75,268,271].

### 7.1. SRS—Skin Penetration Studies

To study skin penetration, it is preferable to have a formulation (substance)-related Raman band in the ≈1700–2820 cm^−1^ spectral region, where skin does not have distinct bands [192]. The chemical alteration of substances by replacing hydrogen with heavier deuterium is often used to shift the FP Raman bands toward higher wavenumbers within the desirable region (≈1700–2820 cm^−1^) [269,272]. SRS spectra are usually characterized by a low non-resonantly excited fluorescence background [273], which can be further minimized by modulation techniques to increase the signal contrast [274]. Thus, SRS serves as an advanced “tracking specific Raman band” analytical method, as described above. 

**Figure 8:**
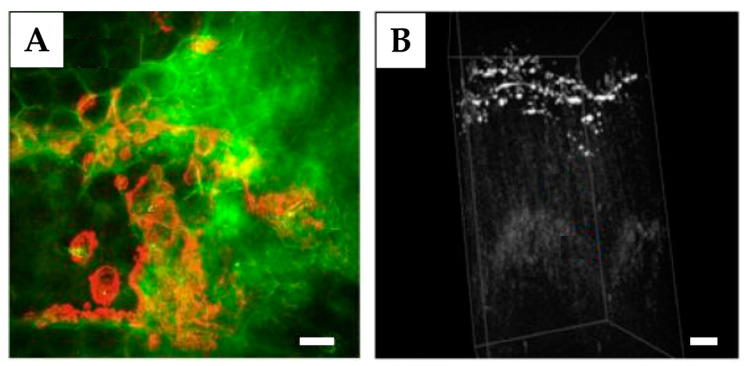
Representative SRS (**A**) and OCT (**B**) images of skin penetration. (**A**): Deposition of the ruxolitinib (red) in the SC of mouse skin (green) ex vivo measured with SRS (“Stokes” beam at 803 nm to target lipids at 2845 cm^−1^ and “pump” beam at 845 nm to target the nitrile stretch of ruxolitinib at 2220 cm^−1^), reprinted with permission from Ref. [279]; (**B**): Penetration of nanodiamonds into mice skin ex vivo (white areas with strong scattering show the presence of nanodiamond clusters in the epidermis) measured with OCT (excitation at 930 nm), adopted under CC BY 4.0 license from Ref. [102]. Scale bar: 20 µm (**A**), 150 µm (**B**).

The ex vivo imaging of the penetration depth profile of deuterated ibuprofen [275,276], ketoprofen [276], lidocaine hydrochloride, loxoprofen sodium [277], caffeine [278], oleic acid-d_34_ [267], ruxolitinib, deuterated betamethasone dipropionate (BMDP-D) [279], dexamethasone [280], deuterated water [281], 4-cyanophenol [282], DMSO, and retinoic acid [268] in skin was determined using SRS. A representative image of the penetration of the drug ruxolitinib into mouse skin ex vivo is shown in Figure 8A. Here, the “Stokes” beam at 803 nm targets lipids at 2845 cm^−1^ and the “pump” beam at 845 nm targets the nitrile stretch of ruxolitinib at 2220 cm^−1^ (both excitation wavelengths lie in the “skin optical transparent window-I” (Figure 3)). The in vivo SRS imaging of mammalian skin constituents and topically applied formulations is possible [283,284,285,286] and very prospective, but is not considered due to the high cost and complexity of the equipment and its limitations for safe application on human skin [273]. 

### 7.2. SRS—Advantages, Limitations, and Applied Substance Requirements

The main merits of SRS microscopy include rapid real-time label-free application and the combination of high chemical sensitivity and molecular specificity with high-spatial-resolution imaging. The limitations are the high cost of the equipment (tunable pulse lasers are required) and the restricted amount of investigated substances due to the strong band overlap with the skin bands (or the need for their deuteration). 

The most important requirement for topically applied substances is the presence of at least one Raman band that does not overlap with the skin bands. Therefore, it is preferable to have a substance-related Raman band in the ≈1700–2820 cm^−1^ spectral region, where skin does not have distinct bands. The overlap of the Raman bands of the substance and skin reduces the detection sensitivity.

## 8. Optical Coherence Tomography (OCT)

OCT is a 3D optical imaging method, whose working principle is based on low coherence interferometry depth-scans [287]. The probe beam light reflected from the areas within the sample (“sample arm”) and the reference mirror (“reference arm”) creates an interferometric intensity image, which is recorded by the detector. Scanning the probe beam with a galvo scanner and the “reference arm” moving the reference mirror provides 3D imaging [288]. The image intensity characterizes the efficiency of reflectance in the “sample arm”, which is increased in regions of high density. The higher the sample heterogeneity (density variation), the higher the contrast of the OCT images. Excitation is performed using a low-coherence broadband light source with wavelengths in the “skin optical transparent window-I, -II, or -III” [221], which have the highest penetration into the skin (Figure 3). The working principle of the OCT is described in detail elsewhere [288,289].

OCT is a common in vivo imaging modality used in skin physiology and dermatology [110,290,291,292,293], typically providing an axial resolution of 5–15 µm down to 1–2 mm [294]. However, modern OCT devices have a higher resolution of <2 µm, achieved through various technical improvements to conventional OCT [295,296,297,298]. 

### 8.1. OCT—Skin Penetration Studies

OCT is not suitable for penetration studies of non-particulate formulations because the changes induced by the formulation in the optical properties of the skin are too small to be detected [110]. On the other hand, particulate formulations with different absorption and backscattering properties contrast with skin constituents and can, therefore, be visualized in the skin with OCT. The penetration and accumulation of calcium carbonate particles [100] and vaccine-loaded carriers [299], nanodiamonds ex vivo [102], as well as silica/gold (core/shell) nanoshells [94,294] and titanium dioxide nanoparticles in vivo [300] have been determined in the skin using OCT. A representative image of the penetration of nanodiamonds (100 nm) into mice skin ex vivo is shown in Figure 8B. The imaging capability of OCT is increasingly being used to study and control microneedle-induced intradermal and transdermal drug delivery in the skin [159,301,302]. 

### 8.2. OCT—Advantages, Limitations, and Applied Substance Requirements

The strengths of OCT in in vivo skin penetration research are its non-invasiveness, fast image acquisition, high screening depth (1–2 mm), and ability to provide high-quality images of skin morphology and particulate formulation (its target substances) in the µm size range. The main disadvantages are the inability to visualize non-particulate substances. The light contrast and the resolution are only sufficient for visualizing particle agglomerates, which is a limitation of the OCT method in penetration studies.

Only particulate substances with absorption and scattering properties different from, skin constituents can be contrasted and visualized in the skin with OCT, which fulfils the main requirement. 

## 9. Conclusions and Future Prospects

The manuscript provides an overview of the optical methods—FCLSM, RCLSM, 2PT-(AF, FLIM, SHG, CARS), 3PM, CRM, SERS, SRS, and OCT—that are most commonly used for the quantitative and qualitative determination of the penetration depth, visualization of deposition, and penetration pathways of vehicles, APIs, FCIs, or contaminants into skin in vivo and non-invasively. These optical methods can also be used in the analysis of skin penetration ex vivo. The requirements for the substances’ optical properties to determine their presence in the skin using certain optical methods are presented. Imaging techniques can visualize the deposition of substances in the SC, viable epidermis, hair follicles, sweat gland ducts, furrows, and wrinkles. Table 1 summarizes the advantages and limitations, and represents a rough comparison of the reviewed optical methods. 

The need for non-invasive, fast, and high-quality skin 2D/3D imaging with chemical contrast information is driving the development of new advanced multimodal devices. The integration of different optical methods into an advanced multimodal device that combines the important advantages of the individual methods (Table 1) will considerably expand the technical possibilities, and has strong prospects for skin research in general and the penetration of target substances into skin in particular. Thus, the integration of non-invasive methods that allow fast image acquisition in a large skin area (advantages of CLSM and OCT) with highly sensitive methods that provide chemical information (advantages of CRM, SERS, SRS, and 2PT-CARS) and high-quality multimodal imaging (advantages of 2PT-AF, 2PT-FLIM, SHG, and 3PT) are expected to be available in the future. This will expand our knowledge on penetration depth and pathways, and allow us to visualize and determine the effect of applied formulations (target substances) on the skin under native and non-destructive in vivo conditions, including chemical and morphological changes caused by the treatment, which are important for dermatology, cosmetology, and pharmacy. The use of non-invasive optical methods in vivo may also reduce the number of invasive tests performed on animal skin. The only disadvantage is a significant increase in the cost of such devices. 

## Figures and Tables

**Table 1:** Possibilities and technical characteristics of the optical methods used in vivo/ex vivo to determine skin penetration.

Optical Method		Imaging Method (2D/3D)	Screening Depth, µm	Lateral/Axial Resolution	Penetration of Non-Particulate Substances	Penetration of Particulate Substances	Possibility to Quantify the Substances Used
**FCLSM**	in vivo	Yes	≈100 µm	<1 µm/ <5 µm	Yes ^1,2^ (low sensitivity)	Yes ^1,2^ (low sensitivity)	Yes [73]
	ex vivo	Yes					
**RCLSM**	in vivo	Yes	≈150 µm		No	Yes (low sensitivity)	No
	ex vivo	Yes					
**2PT-AF**	in vivo	Yes	≈150 µm	<1 µm/ <2 µm	Yes ^2^ (low sensitivity)	Yes ^2^ (low sensitivity)	No
	ex vivo	Yes					
**2PT-FLIM**	in vivo	Yes			Yes ^3^ (high sensitivity)	Yes ^3^ (high sensitivity)	
	ex vivo	Yes					
**SHG**	in vivo	Yes			No	Yes (high sensitivity)	
	ex vivo	Yes					
**2PT-CARS**	in vivo	Yes			Yes (low sensitivity) ^4^	Yes (low sensitivity) ^4^	
	ex vivo	Yes					
**3PT**	in vivo	Yes	<900 µm	<1 µm/ <2 µm	No	No	No
	ex vivo	Yes					
**CRM**	in vivo	No ^5^	≈50 µm	<5 µm	Yes (high sensitivity)	Yes (high sensitivity)	Yes [207,209]
	ex vivo	Yes					
**SERS**	in vivo	No ^5^	≈50 µm	<5 µm	No ^6^	Yes ^7^ (high sensitivity)	No
	ex vivo	Yes					
**SRS**	in vivo	Yes	≈150 µm	<2 µm	Yes (high sensitivity) ^4^	Yes (high sensitivity) ^4^	Yes [160]
	ex vivo	Yes					
**OCT**	in vivo	Yes	<2 mm	<2 µm/ <5 µm	No	Yes (low sensitivity) ^8^	No
	ex vivo	Yes					

^1^ The applied substances should be inherently fluorescent or covalently bound with a fluorescent dye; ^2^ The applied substances should have intense AF whose intensity exceeds skin AF; ^3^ The applied substances should have AF lifetimes that are different from the AF lifetimes of the skin; ^4^ Sensitivity will be higher for substances whose Raman spectrum do not overlap with the skin spectrum; ^5^ Only 1D (*z*-stack point measurements) imaging is possible; ^6^ Non-particulate substances can be identified with SERS-labeled detection microneedles or with TERS (low-invasively on tape strips); ^7^ Only the detection of SERS-active nanostructures is possible; ^8^ The higher the density of target substances, the higher the contrast of OCT images.

## Abbreviations

This section contains a list of all abbreviations used in the main text.

2PT	two-photon tomography
3PT	three photon tomography
AF	autofluorescence
APIs	active pharmaceutical ingredients
ATR-FTIR	attenuated total reflectance Fourier-transform infrared
BMDP-D	deuterated betamethasone dipropionate
CARS	coherent anti-Stokes Raman scattering
CLSM	confocal laser scanning microscopy
CRM	confocal Raman micro-spectroscopy
DMSO	dimethyl sulfoxide
EPR	electron paramagnetic resonance
FCIs	functional cosmetic ingredients
FCLSM	fluorescence confocal laser scanning microscopy
FITC	fluorescein 5-isothiocyanate
FLIM	fluorescence lifetime imaging
FCLSM	fluorescence confocal laser scanning microscopy
FP	fingerprint (400–2200 cm^−1^ spectral region)
FTIR-PAS	Fourier-transform infrared photoacoustic spectroscopy
HWN	high wavenumber (2500–4000 cm^−1^ spectral region)
LDA	linear discriminant analysis
MPT	multi-photon tomography
NAD(P)H	nicotinamide adenine dinucleotide phosphate
NMF	non-restricted multiple least squares fit
NMF molecules	natural moisturizing factor molecules
NNMF	non-negative matrix factorization
OCT	optical coherence tomography
PCA	principal component analysis
PCA	3-(Carboxy)-2,2,5,5-tetramethyl-1-pyrrolidinyloxy
PLSR	partial least squares regression
RCLSM	reflectance confocal laser scanning microscopy
RCM	reflectance confocal microscopy
RCLSM	reflectance confocal laser scanning microscopy
RS	Raman spectroscopy
SC	stratum corneum
SERS	surface-enhanced Raman scattering
SHG	second-harmonic generation
Skin-PAMPA	skin parallel artificial membrane permeability assay
SRS	stimulated Raman scattering
TEMPO	2,2,6,6-Tetramethylpiperidin-1-yl)oxyl
TED-FTIR	thermal emission decay–Fourier-transform infrared
TERS	tip-enhanced Raman spectroscopy
TEWL	transepidermal water loss
THG	third-harmonic generation
tMCR-ALS	tailored multivariate curve resolution–alternating least squares
UV/VIS	ultraviolet/visible (light)
ω_1PEF_	frequency of one-photon-excited fluorescence
ω_2PEF_	frequency of two-photon-excited fluorescence
ω_3PEF_	frequency of three-photon-excited fluorescence
ω_aS_	frequency of anti-Stokes scattering light
ω_p_	frequency of probe laser beam
ω_R_	frequency of Rayleigh scattering light
ω_S_	frequency of Stokes scattering light
